# COVID-19-Related Rhino-Orbital-Cerebral Mucormycosis With Angioinvasion: A Lethal Sequelae

**DOI:** 10.7759/cureus.40483

**Published:** 2023-06-15

**Authors:** Nur Syazwani Redzuwan, Andrea Lillianne Barr Kumarakulasinghe, Wan Mariny W Md Kasim, Safinaz Mohd Khialdin

**Affiliations:** 1 Ophthalmology, Universiti Kebangsaan Malaysia Medical Centre, Kuala Lumpur, MYS; 2 Ophthalmology, Hospital Tengku Ampuan Rahimah, Klang, MYS; 3 Ophthalmology, Hospital Serdang, Kajang, MYS

**Keywords:** central retinal artery occlusion (crao), angioinvasion, steroid therapy, orbital mucormycosis, orbital apex syndrome, rhino-orbital-cerebral, covid-19, mucormycosis

## Abstract

A middle-aged man with multiple comorbidities including uncontrolled diabetes mellitus presented with shortness of breath and lethargy for six days. He was treated for COVID-19 pneumonia, requiring high cumulative steroid therapy. After 15 days of treatment, he developed right orbital apex syndrome with central retinal artery occlusion secondary to invasive mucormycosis. The infection progressed rapidly despite aggressive medical treatment, systemic anti-fungal therapy along with local transcutaneous retrobulbar amphotericin B injection. We report our battle in fighting this vicious disease. Judicious use of immunomodulators in COVID-19 treatment and close monitoring is crucial, especially in high-risk patients.

## Introduction

Mucormycosis is a rare life-threatening fungal infection caused by fungi that belong to the order *Mucorales* [1]. The most virulent organism of this group is the *Rhizopus* spp. Other genera with mucormycosis-causing species are *Mucor*, *Cunninghamella*, *Apophysomyces*, *Lichtheimia* (formerly *Absidia*), *Saksenaea*, *Rhizomucor*, and other species [1,2]. These fungi are ubiquitous. Human beings are exposed to fungal spores every day. However, in immunocompromised states, it causes opportunistic infections. This was witnessed during the COVID-19 pandemic, where an unprecedented increase in the number of mucormycosis was observed [3]. This was postulated to be due to COVID-19 patients being treated with steroids rendering them vulnerable to this virulent organism. This opportunistic infection is associated with high morbidity and mortality rate.

## Case presentation

A 58-year-old Malay male with underlying diabetes mellitus, hypertension, dyslipidemia, and ischemic heart disease presented with a six-day history of shortness of breath and generalized malaise. On presentation, he was febrile (38°C), hypoxic with an oxygen saturation of 80%, and hyperglycemic with a random blood glucose of 15.9mmol/l with no metabolic acidosis or ketosis. Otherwise, his blood pressure and heart rate were normal. A polymerase chain reaction from a nasopharyngeal swab was positive for the SARS-CoV-2 virus. He was treated for COVID-19 pneumonia category 4A and uncontrolled diabetes mellitus. He was started on oral favipiravir 1600mg twice daily on day one followed by 800mg twice daily for a total of seven days, intravenous methylprednisolone 100mg daily, and supplemental oxygen via a face mask. Glycemic control was optimized with insulin therapy.

On day 16 of illness, at a cumulative steroid dose of 2g, he developed sudden drooping of the right eyelid. On further history, there was also reduced right eye vision with pain on eye movements associated with a headache for one day. There was no eye redness, eye discharge, or any other associated symptoms.

On examination, the patient appeared lethargic and tachypnoeic, requiring two liters/minute of oxygen on nasal prongs. The right reverse relative afferent pupillary defect was positive. There was a loss of light perception in all quadrants of the right eye, whereas the left eye’s visual acuity was 6/30. No obvious proptosis was present. Right partial ptosis was noted with limitations of ocular motility in all directions. Anterior segments of both eyes were unremarkable except for the presence of anisocoria. The left pupil was 4mm while the right was 1mm. Both eyes were digitally normotensive. However, the right globe was significantly tender on palpation. (Intraocular pressure was not taken due to COVID restrictions.) The right optic disc was pink with a cup disc ratio of 0.3. There was retinal whitening at the posterior pole with an apparent cherry-red spot. The left eye fundus examination was unremarkable with no diabetic retinopathy seen bilaterally.

**Figure 1:**
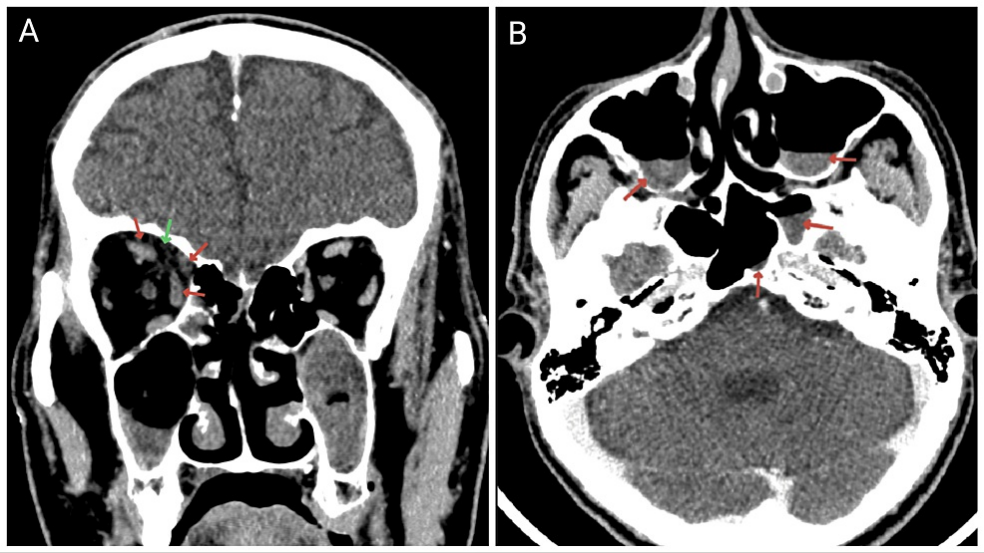
CECT showing (A) orbital fat stranding (green arrow) with swollen superior rectus, medial rectus, and superior oblique muscles (red arrows) and (B) mucosal thickening of maxillary and sphenoid sinuses (red arrows)

A right orbital apex syndrome with central retinal artery occlusion diagnosis was made. Urgent contrast-enhanced computed tomography (CECT) of the brain and orbit revealed right orbital fat stranding, swollen right superior rectus, medial rectus, and superior oblique muscles, with mucosal thickening of bilateral maxillary, ethmoid, and sphenoid sinuses. Cavernous sinuses and orbital apices were symmetrical bilaterally (Figure 1).

Right orbital apex syndrome secondary to invasive fungal sinusitis was suspected due to the high cumulative steroid therapy and uncontrolled diabetes mellitus in this patient. With the steroid administration, the glucose level fluctuated and ranged from 10mmol/L to 19mmol/L in ward. Urgent diagnostic nasal endoscopy revealed pale mucosa with fungal debris at the right middle turbinate. Intravenous amphotericin B was initiated. Unfortunately, due to renal impairment, a renal-adjusted dose of 0.5mg/kg daily of amphotericin B had to be administered.

An emergency endoscopic sinus surgery was performed with extensive debridement of the involved sinonasal mucosa. Intra-operatively, there were multiple areas of necrotic patches and unhealthy mucosa. The right lamina papyracea was removed, periorbital fat was debrided, and right orbital decompression was done.

Extensive colonization of *Rhizopus oryzae* was seen as evidenced by the tissues cultured from the right osteomeatal complex, right anterior and posterior ethmoid, right middle turbinate, right uncinate, and right orbital fat. There was also *Aspergillus niger* growth obtained from the left posterior ethmoid sinus. The tissues which were taken from the right sphenoid and left maxillary sinuses grew both of the organisms.

Prior to the emergency operation, a rapid right eye deterioration was observed within 12 hours of the initial ocular examination as there was progression to obvious axial proptosis and complete ptosis with total ophthalmoplegia. Blackish discoloration of the skin was seen over the medial part of the right upper and lower lids. There were conjunctival injections and chemosis. Fundus examination showed a right swollen hyperaemic disc with retinal whitening and a cherry-red spot at the posterior pole.

Due to the fast deterioration in the ocular examination, urgent contrast-enhanced magnetic resonance imaging (CE-MRI) was requested to look for an extension of the disease. CE-MRI showed worsening right orbital inflammatory changes with an intracranial extension, evidenced by pachymeningeal dural enhancement at the right temporal lobe.

**Figure 2:**
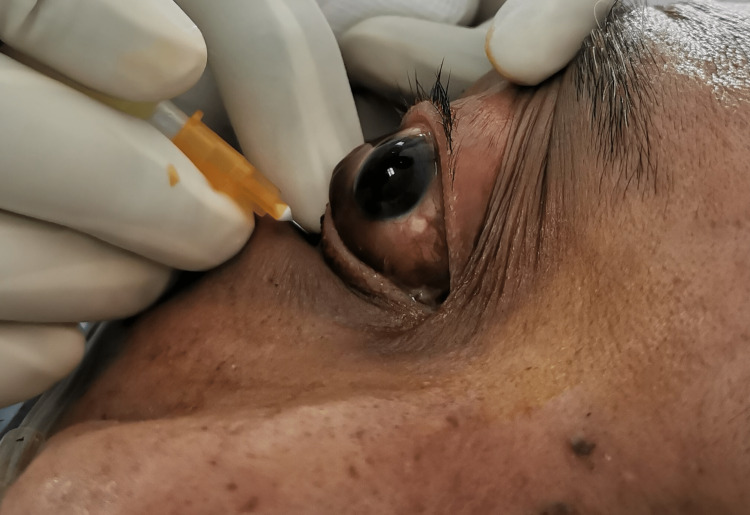
Transcutaneous retrobulbar amphotericin B injection

One day after the sinonasal debridement, he was given transcutaneous retrobulbar amphotericin B 3.5mg/ml daily for three days (Figure 2).

**Figure 3:**
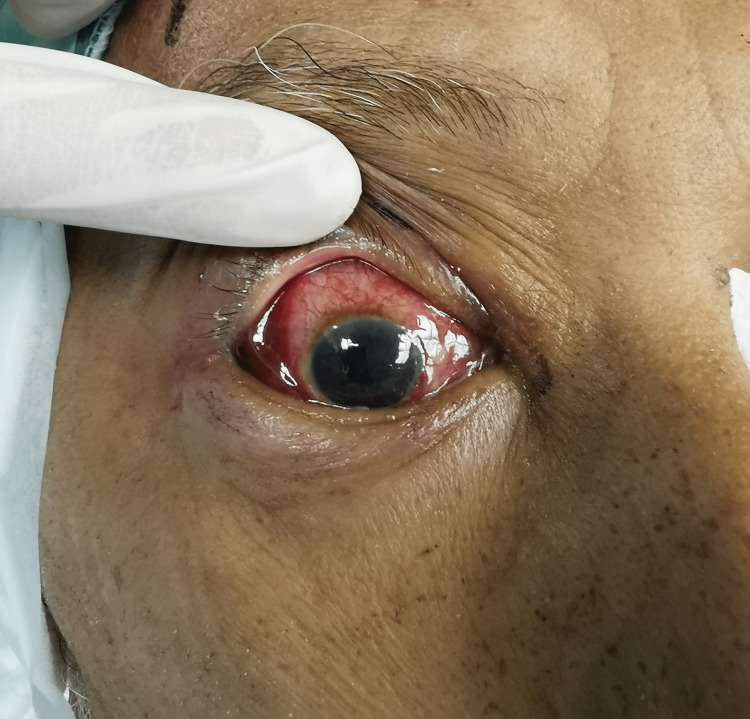
Worsening conjunctival injection with corneal edema

Despite three consecutive local amphotericin B injections, the ocular condition deteriorated. Proptosis worsened with lagophthalmos and massive vitreous hemorrhage obscuring the optic disc and posterior pole was observed. Signs of anterior segment ischemia were also observed as evidenced by conjunctival injection and corneal edema (Figure 3).

A combined sinonasal debridement and orbital exenteration were performed following failed attempts at medical treatment.

**Figure 4:**
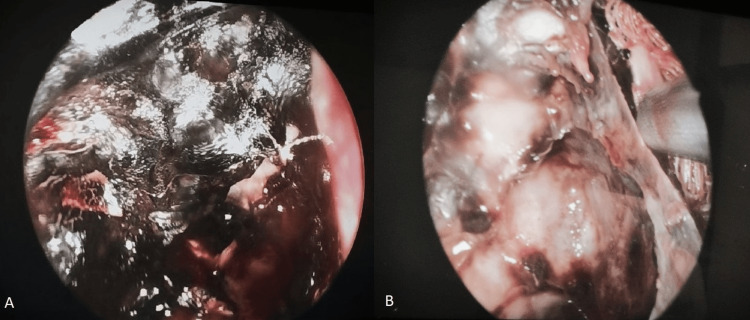
Nasal endoscopic images showing (A) extensive necrotic tissues of paranasal sinuses and (B) pale unhealthy mucosa on nasal endoscopy

Examination under anesthesia, right orbital exenteration with nasal toileting, and debridement were performed. Extensive involvement of bilateral paranasal sinuses was seen as necrotic tissues were being debrided (Figure 4A). Unhealthy pale mucosa was also apparent (Figure 4B).

**Figure 5:**
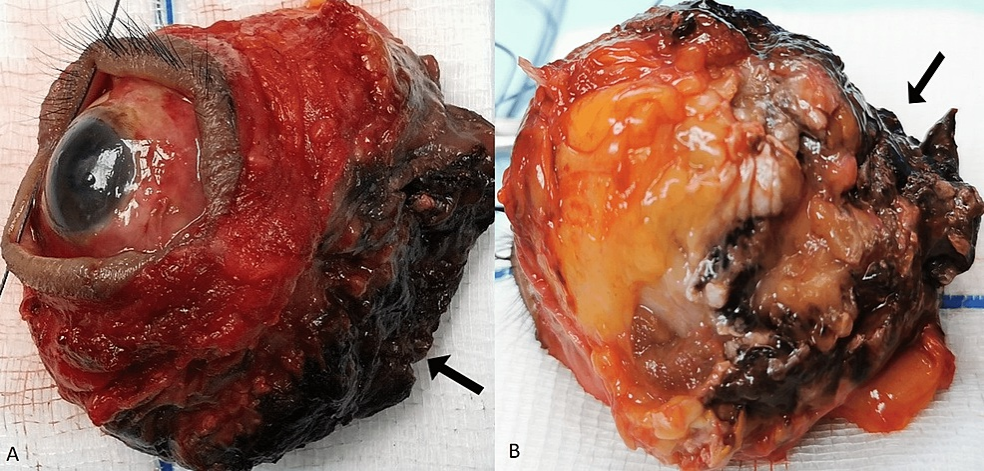
Blackish discoloration of posterior one-third of exenterated orbital content (black arrows). (A) Medial view (black suture was used to tag the lateral part of the eye) and (B) superior view

**Figure 6:**
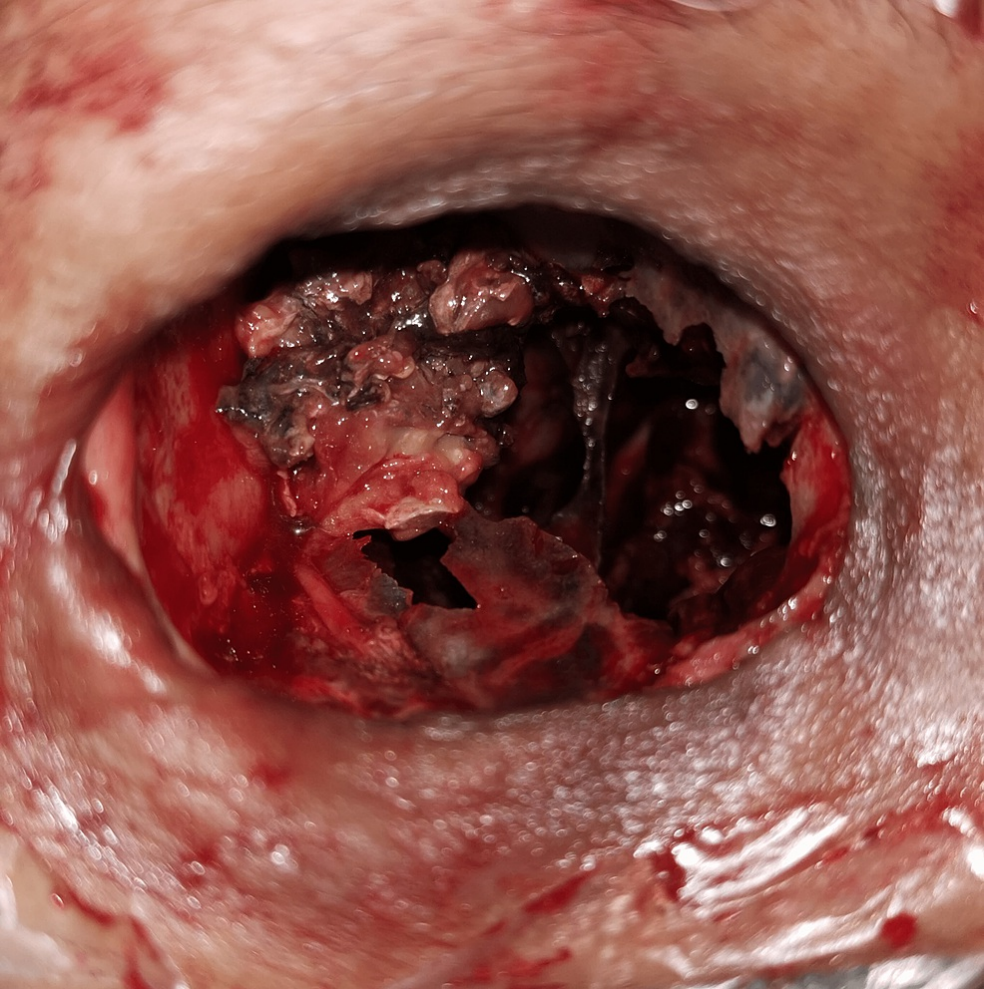
Unhealthy orbital tissue with involvement of superior, medial, and inferior right orbital walls extending to paranasal sinuses was seen intra-operatively

Necrotic tissues were seen involving the posterior one-third of the orbital content (Figure 5A, 5B). There was also the involvement of the superior, medial, and floor of the right orbital walls (Figure 6).

**Figure 7:**
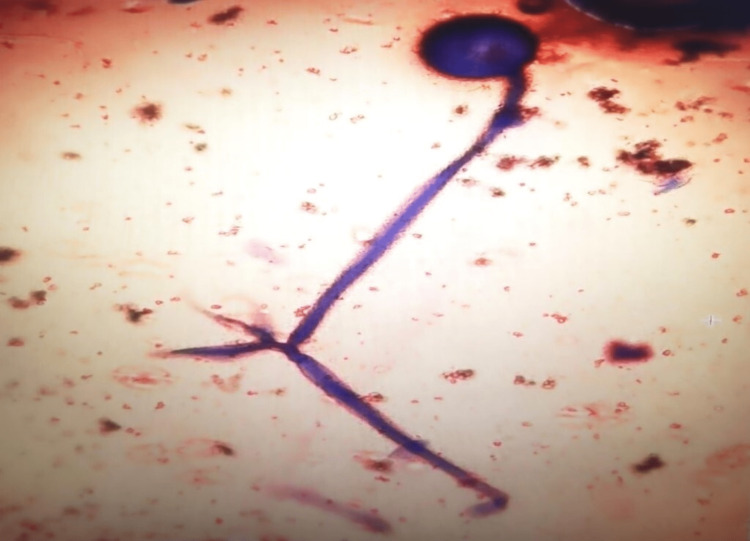
Histopathology showed broad, non-septate hyphae of Mucor spp. with wide-angle branching

Histopathological examination of the orbit and surrounding soft tissues showed extensive infiltration of numerous fungal bodies with necrotic tissue and neutrophilic exudates. The fungal bodies displayed broad ribbon-like hyphae forms suggestive of *Mucor* spp. (Figure 7) and were seen infiltrating recti muscles, blood vessels, and nerve bundles extending up to the sclera. Periodic acid-Schiff and Grocott’s methenamine silver stains were both positive for the fungal organisms. Unfortunately, he developed multiple systemic complications and eventually succumbed to his illness on day three post-operation.

## Discussion

During the COVID-19 pandemic, a rapid surge in the number of cases of rhino-orbital mucormycosis was noted especially in India [3]. Rhino-orbital mucormycosis is a progressive and potentially lethal angio-invasive disease predominantly affecting immunocompromised individuals. In the era of the coronavirus pandemic, indiscriminate use of systemic steroids, pre-existing diseases such as diabetes mellitus, and systemic immune alterations caused by the virus itself are among the major factors contributing to the invasive fungal infection [4,5].

Although the treatment approach in treating fungal sinusitis is generally straightforward, the best way to manage infected orbital tissue remains a challenge. The role of exenteration in the management of invasive orbital mucormycosis has been a source of significant debate for decades. Although progressive orbital disease warrants exenteration, there has been a trend in the literature toward a less aggressive approach. There are three treatment options for infected orbital tissue: exenteration, conservative debridement with irrigation, and transcutaneous retrobulbar Amphotericin B [6]. Local injection of liposomal amphotericin B in the retroorbital space is an option to reduce the risk of radical exenteration without compromising survival [6].

Kalin-Hajdu et al. proposed an algorithm for the management of orbital mucormycosis based on clinical examination and orbital soft tissue imaging. The role of tissue debridement and transcutaneous retrobulbar amphotericin B is considered in early orbital infections, whereas orbital exenteration is performed when the disease becomes severe [6]. The rationale of this algorithm is to attain local control of the infection without unnecessary radical intervention in mild cases or delay surgical treatment in severe conditions.

In an advanced stage of rhino-orbital-cerebral mucormycosis (ROCM), central retinal artery occlusion may develop as it is a rare complication of ROCM with an incidence of 16-20%. Direct infiltration of the central retinal artery causes arterial occlusion as evident by posterior pole retinal opacification with a cherry-red spot at the macula on fundus examination [7].

Mucormycosis causes blood vessel thrombosis and extensive tissue necrosis due to the angioinvasive property of the fungus. This limits drug penetration to the infected sites which leads to treatment failure. Therefore, debridement of necrotic tissue in combination with anti-fungal therapy is mandatory for patient survival [8].

In our patient, he underwent early sinus debridement along with intravenous amphotericin-B. Unfortunately, a suboptimal dose of the antifungal drug had to be administered due to nephrotoxicity. In view of proptosis, total visual loss with cranial neuropathies along with orbital fat stranding and recti muscles enhancement as demonstrated by the imaging, he was given a series of retrobulbar injections of liposomal amphotericin B once daily for three consecutive days as proposed by the algorithm. This targeted treatment was also a better option at this stage as a higher dose of intravenous amphotericin B was contraindicated.

Previous literature has reported the administration of retro-bulbar antifungal therapy for the treatment of invasive orbital fungal infections with a number of successful globe salvage treatments [9-13].

Unfortunately, in our patient, after three series of retrobulbar amphotericin B administration, there was no improvement, and further deterioration of the orbital disease was noted. Hence, orbital exenteration had to be performed along with repeat sinus debridement. Post-operatively, the management of his systemic conditions was even more challenging, as he developed multiple life-threatening complications.

## Conclusions

The widespread use of corticosteroids and monoclonal antibodies in the treatment of COVID-19 caused an increase in the number of invasive fungal infection cases, especially in those with pre-existing risk factors. As the management of invasive ROCM remains a challenge to clinicians, extra attention and a high index of suspicion of this fatal infection should be given especially to those who are susceptible during the treatment of COVID-19.
